# The Impact of SNP Score on Low-Density Lipoprotein Cholesterol Concentration and Coronary Artery Disease

**DOI:** 10.3390/ijms26052337

**Published:** 2025-03-06

**Authors:** Darius Čereškevičius, Ieva Čiapienė, Ali Aldujeli, Vytautas Zabiela, Vaiva Lesauskaitė, Kristina Zubielienė, Vytautas Raškevičius, Diana Žaliaduonytė, Ramūnas Unikas, Robertas Pranevičius, Ignas Simanauskas, Giedrė Bakšytė, Abdonas Tamošiūnas, Dalia Lukšienė, Gintarė Šakalytė, Vacis Tatarūnas

**Affiliations:** 1 Institute of Cardiology, Lithuanian University of Health Sciences, Sukileliu 15, 50103 Kaunas, Lithuania; darius.cereskevicius@lsmu.lt (D.Č.); ieva.ciapiene@lsmu.lt (I.Č.); ali.aldujeli@lsmu.lt (A.A.); vytautas.zabiela@lsmu.lt (V.Z.); vaiva.lesauskaite@lsmu.lt (V.L.); kristina.zubieliene@stud.lsmu.lt (K.Z.); vytautas.raskevicius@lsmu.lt (V.R.); robertas.pranevicius@lsmu.lt (R.P.); ignas.simanauskas@lsmu.lt (I.S.); abdonas.tamosiunas@lsmu.lt (A.T.); dalia.luksiene@lsmu.lt (D.L.); gintare.sakalyte@lsmu.lt (G.Š.); 2 Department of Cardiology, Kaunas Hospital of the Lithuanian University of Health Sciences, Hipodromo 13, 45130 Kaunas, Lithuania; diana.zaliaduonyte@lsmu.lt; 3 Department of Cardiology, Lithuanian University of Health Sciences, Eivenių 2, 50009 Kaunas, Lithuania; ramunas.unikas@lsmu.lt (R.U.); giedre.baksyte@lsmu.lt (G.B.)

**Keywords:** SNP score, low-density lipoprotein cholesterol, coronary artery disease, PRS, hypercholesterolemia

## Abstract

Hypercholesterolemia, characterized by elevated levels of low-density lipoprotein cholesterol (LDL-C), along with inflammation, is a well-known risk factor for developing atherosclerosis and coronary artery disease (CAD). Many patients with hypercholesterolemia may carry inherited genetic variants that are not part of the commonly recognized mutations in the *LDLR*, *APOB*, *LDLRAP1*, and *PCSK9* genes. These genetic variants may have cumulative effects that contribute to increased LDL-C levels and CAD development. The polygenic risk score (PRS) may provide an essential tool for evaluating an individual’s genetic predisposition to these conditions. This pilot study aimed to investigate the impact of the PRS calculated from specific single nucleotide polymorphisms (SNPs) associated with LDL cholesterol (LDL-C)—namely, *CELSR2 rs629301*, *APOB rs1367117*, *ABCG8 rs6544713*, *LDLR rs6511720*, *APOE rs429358*, and *rs7412*—on LDL-C levels in both healthy individuals with elevated LDL-C levels (>2.6 mmol/L) and those diagnosed with ST-segment elevation myocardial infarction (STEMI). A total of 61 healthy individuals with high LDL-C levels (>2.6 mmol/L) and 93 STEMI patients were selected for the study. The High-Resolution Melting Polymerase Chain Reaction (HRM PCR) method was adopted and sequencing techniques were employed to identify the specific single nucleotide polymorphisms (SNPs) of interest. The patient group exhibited a PRS of 0.824 (with a range of −0.62 to 1.174) compared to 0.674 (range: −0.176 to 0.974) in healthy individuals, indicating a higher genetic predisposition to elevated LDL-C levels (*p* = 0.001) in patients. Interestingly, patients had lower LDL-C concentrations than healthy individuals. Additionally, a more significant number of patients were past smokers and statin users. The PRS calculations revealed that patients with a higher PRS had increased odds of experiencing an MI, with an odds ratio of 12.044 (95% confidence interval: 1.551–93.517, *p* = 0.017). Similarly, smokers showed even higher odds, with an odds ratio of 24.962 (95% CI: 7.171–86.890, *p* < 0.001). Among healthy individuals, those with a higher PRS had increased odds of having an LDL-C concentration greater than 4.9 mmol/L (odds ratio: 20.391, 95% CI: 1.116–358.486, *p* = 0.039). However, no significant association was found between the PRS and LDL-C levels in the patient group during hospitalization (*p* = 0.782). This pilot study shows that PRS can be employed to evaluate the risk of MI and to estimate concentrations greater than 4.9 mmol/L LDL-C in healthy individuals.

## 1. Introduction

Coronary artery disease (CAD) has emerged as a significant public health problem, affecting around 126 million individuals worldwide [1,2]. CAD is primarily caused by atherosclerosis, which results in reduced blood flow through the damaged coronary arteries to the heart muscle, leading to myocardial ischemia. Further progression of atherosclerosis and inflammation can lead to thrombosis and acute coronary syndromes (ACS).

Elevated levels of plasma low-density lipoprotein cholesterol (LDL-C) and inflammation are established risk factors for developing atherosclerosis and ACS [3]. Alongside their physiological function to carry lipids to specific sites in the organism, blood plasma lipoproteins play a crucial role in the development of atherosclerosis [4]. Plasma lipoproteins are a combination of specific proteins and lipids, and are classified into seven classes based on their density, size, lipid composition, and apolipoproteins. Lipoproteins have various destinations and functions within the human body [5]. LDL-C-lowering therapies have consistently been shown to reduce cardiovascular mortality. Therefore, identifying high LDL-C levels in blood plasma and managing them is common practice in preventing CAD today [6].

Both genetic and lifestyle factors determine hypercholesterolemia or high blood plasma lipoprotein cholesterol level status [7,8,9]. Mutations in four genes that encode proteins involved in the breakdown and absorption of LDL (*LDLR*, *APOB*, *LDLRAP1,* and *PCSK9* genes) are responsible for most cases of familial hypercholesterolemia (FH). The LDL receptor binds explicitly to apolipoprotein B (ApoB), which surrounds the LDL particle. The LDL receptor adaptor protein, encoded by the *LDLRAP1* gene, plays a significant role in transporting LDL receptors and attached LDL particles from the cell surface to the cell’s interior, where LDL endocytosis occurs [10]. Proprotein convertase subtilisin/kexin type 9 (PCSK9), encoded by the *PCSK9* gene, regulates the lysosomal degradation of hepatic LDL receptors, thereby modulating LDL uptake [11]. However, mutations of genes associated with monogenic FH are present in only about 40–60% of individuals with a clinical expression of FH. Known genetic factors do not explain the remaining portion [12]. Another significant number of patients with hypercholesterolemia who have undetected mutations may have different genes causing the condition. Some patients may have a mutation in a gene that has not yet been identified, or more likely, the condition is due to the inheritance of multiple genetic variants. Polygenic hypercholesterolemia occurs when the cumulative effect of genetic variants in multiple genes with minor LDL-C boosting properties leads to high LDL-C levels, like levels in patients with monogenic FH [13]. Polygenic hypercholesterolemia is diagnosed using a polygenic risk score (PRS) [14]. There have been 100 loci identified that influence LDL-C levels [15]. The more single nucleotide polymorphisms (SNPs) that elevate LDL-C, the more severe the individual’s hypercholesterolemia phenotype. However, specific genes associated with an LDL-C reduction may mitigate the previously mentioned adverse effects [16]. Overall, 80% of individuals confirmed without *LDLR*, *APOB*, *LDLRAP1*, or *PCSK9* mutations are likely to have polygenic hypercholesterolemia [16]. Specialized methods to identify FH-associated gene variants are expensive or have high setup costs. In 2015, Futema et al. showed that there are primarily 6 SNPs that can help to distinguish FH patients from healthy subjects. These SNPs are *CELSR2* rs629301, *APOB* rs1367117, *ABCG8* rs6544713, *LDLR* rs6511720, *APOE* rs429358, and rs7412 [17]. Further studies have shown that the PRS could explain hypercholesterolemia in patients without pathogenic variants in FH-causative genes [18]. The PRS is a way to summarize the combined influence of multiple gene variants on a patient’s phenotype [19]. In this pilot study, we aimed to investigate how the PRS [17] may affect LDL-C levels in both healthy individuals with elevated LDL-C levels (>2.6 mmol/L) and those experiencing ST-segment elevation myocardial infarction (STEMI).

## 2. Results

The age did not differ between members of the comparison group (at the time of enrolment into the study) and the patient group at the time of hospitalization (*p* = 0.259). See Table 1 for the characteristics of each group. There was no significant difference in the sex distribution between the groups (*p* = 0.625). The patients had lower LDL-C concentrations at enrolment and were more frequently past smokers and statin users. In contrast, subjects in the comparison group exhibited a higher prevalence of hypertension at the time of enrolment (*p* = 0.004).

All seven SNPs selected for analysis were successfully genotyped in the patient and comparison groups. The frequencies of these SNPs used in calculating the PRS are detailed in Table 2.

**Figure 1:**
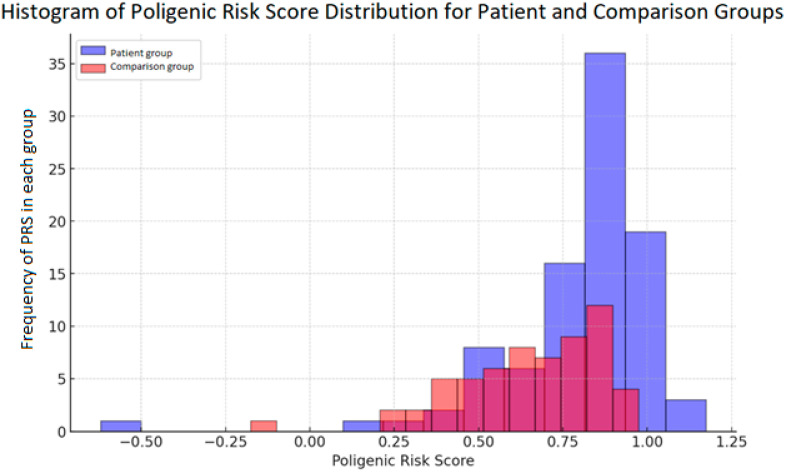
PRS distribution in patient and comparison groups representing counts of individuals.

Regarding the genetic risk, the median LDL-C SNP PRS for subjects in the comparison group was 0.674 (−0.176 to 0.974). In contrast, subjects in the patient group exhibited a significantly higher median LDL-C SNP score of 0.824 (−0.62 to 1.174), indicating a higher genetic predisposition to elevated LDL-C levels (*p* = 0.001). The distribution of the PRS across both groups is depicted in Figure 1, where the separation between the comparison and patient groups is apparent. The regression analysis revealed that both the PRS and smoking significantly increased the odds of developing MI. Individuals with a higher PRS (odds ratio: 12.044 (95% CI: 1.551–93.517, *p* = 0.017) or smokers (odds ratio: 24.962 (95% CI: 7.171–86.890, *p* < 0.001) demonstrated markedly increased odds of experiencing MI.

Further regression analysis revealed a significant link between LDL-C levels and the PRS within the comparison group. Individuals with a higher PRS demonstrated increased odds of having an LDL-C concentration greater than 4.9 mmol/l (odds ratio: 20.391, 95% CI: 1.116–358.486, *p* = 0.039). No significant link was found between the PRS and LDL-C levels in the patient group during hospitalization (*p* = 0.782).

## 3. Discussion

This pilot study compared the PRS in patients with MI to individuals with elevated LDL-C levels (>2.6 mmol/L), identified through epidemiological screening. Notably, only three individuals in the comparison group experienced cardiovascular events.

Epidemiological studies, such as the Framingham Heart Study, have been instrumental in understanding atherosclerotic cardiovascular disease (ASCVD). This study established traditional risk factors, including smoking, hypertension, and elevated plasma cholesterol, as key contributors to ASCVD [20]. Even with the effective management of these risk factors, their influence on the progression of the disease persists [21]. Recent findings from the Framingham Heart Study also highlight genetic variations as significant contributors to cardiovascular risk [20].

Our study revealed that patients were more likely to be former smokers, to have comorbidities, such as diabetes mellitus or renal insufficiency, and to have a family history of CVD compared to those in the comparison group. Interestingly, individuals in the comparison group had hypertension more frequently than patients. The patients exhibited lower LDL-C concentrations than comparison group individuals, potentially due to the acute phase of MI and lipid-lowering therapy. A significant portion of patients (31.2%) were already hospitalized while on statins, whereas only 5% of subjects in the comparison group were statin users. The risk factors observed in the patient group strongly highlight the consensus that multiple elements play a significant role in the onset of cardiovascular disease [22,23].

LDL-C values decrease significantly during the acute phase of ACS [24]. Fluctuations in the serum lipid profile are observed after STEMI; total cholesterol, LDL-C, and HDL-C typically decrease in the first few days following admission with STEMI [25], but triglyceride levels may increase [26]. Post-MI statins are a cornerstone of secondary prevention, reducing major adverse cardiac and cerebrovascular events (MACCEs) by inhibiting cholesterol synthesis [27]. The current European Society of Cardiology guidelines recommend treating ACS patients with high doses of lipid-lowering drugs [28]. However, individual responses to such treatments can vary significantly [23]. Lipid metabolism and cholesterol gene expression are altered during the acute MI phase. Lymphocytes in patients with acute MI may accumulate cholesterol inconsistently with plasma LDL-C levels, potentially influencing disease progression [29]. LDL-C alone may not fully represent the proatherogenic state, as specific fractions, such as small, dense LDL-C (sdLDL-C), also contribute. While we could not measure sdLDL-C in this study, prior research links sdLDL-C to the progression of coronary artery lesions [30]. The JUPITER trial associated sdLDL-C with coronary heart disease risk, even in patients treated with statins [31], while the AIM-HIGH trial found that sdLDL-C did not predict CVD events in patients with low HDL-C and controlled LDL-C [32].

This study employed a cost-effective combination of genotyping methods to calculate the PRS. HRM was selected as a reliable and economical method, allowing the multiplexing of different amplicons without needing separately labelled probes [33,34,35]. To our knowledge, this is one of the first studies to use HRM to genotype SNPs linked to dyslipidemia in the context of STEMI. The present study establishes that the genetic risk of MI can be quantified by applying a PRS. This score incorporates SNPs associated with dyslipidemia identified in previous research: rs629301, rs1367117, rs429358, rs7412, rs1800562, rs11220462, and rs6511720. The rs629301 variant is a type of cadherin known as cadherin EGF LAG seven-pass G-type receptor 2 (CELSR2). CELSR2 is an atypical cadherin in the plasma membrane and is associated with various human diseases. However, its relationship with lipid levels in blood plasma remains unclear [36]. Our study showed a higher prevalence of rs629301 in patients with STEMI. A study indicated an association between the *CELSR2* T/T risk genotype, elevated ApoE and ApoB levels, and coronary artery stenosis extension [37]. The variant rs1367117 is located within the *APOB* gene. ApoB is a major component of lipoprotein molecules, and its variants are thought to play a role in enhancing the synthesis of LDL-C while decreasing its uptake in the liver. Maternal associations of rs1367117 have been observed with fasting glucose and insulin levels. Therefore, ApoB has shown evidence of its influence on adiposity [38]. The rs429358 and rs7412 are *APOE* variants, producing three major alleles: ε2, ε3, and ε4. A study conducted on the Lithuanian population has demonstrated that these variants are associated with concentrations of LDL-C. Since ApoE interacts with LDL receptors and facilitates the clearance of chylomicron remnants and very low-density lipoprotein (VLDL) cholesterol, it plays a significant role in the normal processing of lipids [39]. Individuals in the comparison group of our study had a lower frequency of rs429358. The rs6511720 variant has been shown to upregulate the expression of LDLR [40]. The function and effects of LDLR were discussed in the Introduction section. The rs1800562 variant causes the HFE protein to be retained in the endoplasmic reticulum rather than transported to the plasma membrane. It has been linked to increased levels of serum ferritin and hemochromatosis [41]. One study has shown that HFE likely downregulates the expression of LDLR [42]. The rs11220462 variant within the *ST3GAL4* gene has been linked to LDL-C during GWAS studies. *ST3GAL4* encodes a member of the glycosyltransferase 29 family, a group of enzymes involved in protein glycosylation. Variants in the *ST3GAL4* gene have been associated with an increased risk of cirrhosis, type 2 diabetes, and CVD, likely by affecting liver enzyme concentrations [43]. However, it is unclear what functional impact this variant has. Thus, in this study, the PRS and smoking significantly increased the odds of developing MI. This aligns with evidence suggesting that a genetic predisposition to elevated LDL-C, as reflected by a higher PRS and traditional risk factors like smoking, contributes to atherosclerosis. This is consistent with the current scientific understanding [32,44].

In this study, higher odds of having LDL-C concentrations >4.9 mmol/L were only significant in the comparison group. This discrepancy is likely due to the acute phase of ACS experienced by subjects in the patient group at enrollment, coupled with the variable responses to treatment (for example, lipid-lowering therapy, as mentioned above, is another option to consider). A comprehensive study, SWEDEHEART, which included detailed data from 63168 statin-naïve patients hospitalized with their first MI, found that high levels of LDL-C at the time of the MI are linked to a lower risk of death from all causes, but a higher risk of experiencing a non-fatal MI during follow-up. However, researchers also noted that these results could reflect a lower age at MI, fewer comorbidities, and the modifiability of LDL-C of studied patients [45]. The fluctuation in cholesterol levels could be explained simply as follows: the more extensive the MI damage, the more significant the decrease in cholesterol during the acute phase, as LDL-C is utilized to repair damaged tissue. This reduction could correlate with cardiac troponin T protein concentrations, as greater troponin T levels indicate a more extensive MI, while LDL-C shows a decrease [24,46]. Despite the limitation of a small sample size, these findings highlight the potential of the PRS to predict MI and elevated LDL-C levels when combined with other risk factors. Using preventive programs, the PRS could be a precise diagnostic tool for assessing cardiovascular risk in high-risk individuals.

Another limitation of this study is that the absence of current smokers in the STEMI group was likely due to the acute medical context of hospitalization. Smokers may have temporarily quit or reduced their smoking due to medical advice or the life-threatening nature of their condition. Moreover, smoking status data were self-reported by patients, which may indicate a desire to change their behavior following a STEMI event. This is further supported by the fact that more than half of the population reported being smokers before the event, suggesting a significant change in behavior or reporting in response to the acute health crisis. This study is also limited by including only STEMI patients, which restricts its applicability to other CAD phenotypes. The sample of healthy subjects was also limited, consisting solely of individuals with LDL-C concentrations of 2.6 mmol/L or greater. To validate and extend our findings, further research with an external validation cohort consisting of larger populations is necessary.

## 4. Materials and Methods

### 4.1. Patient and Comparison Group Samples

The patient group included 93 patients admitted with STEMI between January and November 2021 who underwent invasive angiography accompanied by primary percutaneous coronary intervention (PCI) at the Hospital of the Lithuanian University of Health Sciences in Kaunas Clinics, applying specific exclusion criteria to ensure a homogeneous patient group and minimize confounding factors (Figure 1). Patients with atrial fibrillation or pericardial disease were excluded, as these conditions could interfere with the interpretation of myocardial ischemia on electrocardiograms. Patients with a history of prior coronary artery bypass graft (CABG) surgery were excluded due to potential alterations in coronary anatomy that could affect study outcomes. Those with significant structural heart diseases, such as valvular heart conditions, were also excluded to avoid complications related to non-STEMI-related cardiac dysfunctions. The study excluded patients with severe hepatic, oncological, or lung diseases, renal failure, or a known allergy to contrast media to prevent comorbidities that could independently increase morbidity and mortality risks. Individuals with severe dementia were excluded due to concerns about their ability to provide informed consent and comply with study protocols. Finally, patients with a positive test for COVID-19 were excluded to prevent confounding results due to the potential cardiovascular effects of the virus.

**Figure 2:**
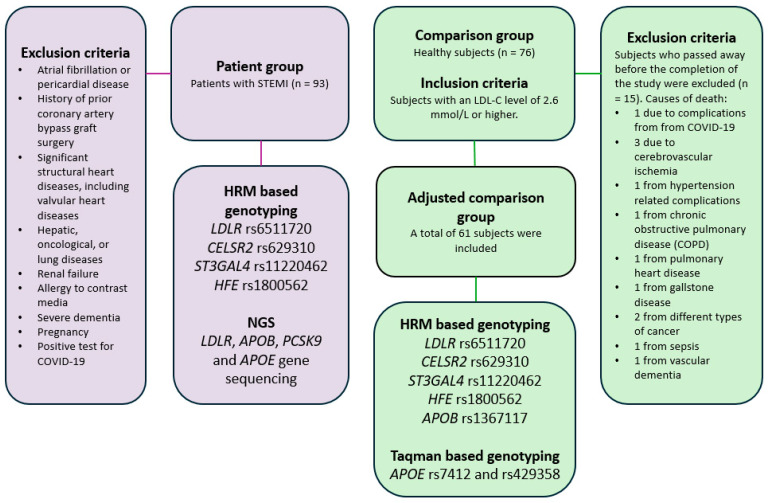
Patient and healthy population sample and analysis methods.

From the International Health, Alcohol and Psychosocial Factors in Eastern Europe (HAPIEE) study conducted in Kaunas, Lithuania [47], 76 subjects with an LDL-C level of 2.6 mmol/L or higher were selected. They formed the comparison group for our study. From 2016 to 2024, 15 individuals in the comparison group passed away and were excluded from the study. The causes of death spanning a variety of conditions were as follows: 1 individual succumbed to complications from COVID-19, 3 individuals died from cerebrovascular ischemia, 1 from hypertension-related complications, 1 from chronic obstructive pulmonary disease (COPD), 1 from pulmonary heart disease, 1 from gallstone disease, 2 from different types of cancer, 1 from sepsis, and 1 from vascular dementia. A total of 61 individuals were finally included in the comparison group. Among these individuals, only two had angina pectoris. Additionally, one subject had cerebral vascular disease. The remaining participants included in the further evaluation had no history of cardiovascular disease, stroke, or diabetes. Subject inclusion and exclusion criteria are presented in Figure 2.

### 4.2. Next Generation Sequencing

Genomic DNA extraction was performed using standard laboratory procedures. The concentration of DNA was determined using a UV spectrophotometer. For library preparation, “Illumina DNA prep” kits (Illumina, San Diego, CA, USA) were used. Libraries were prepared following the manufacturer’s protocols, using 200–500 ng of DNA. A custom panel of Integrated DNA Technologies (IDT) probes (IDT, Coralville, IA, USA) enriched targeted regions. Sequencing was carried out on an Illumina “MiniSeq” system (Illumina, San Diego, CA, USA). Sequencing data were analyzed following the GATK best practice guidelines [48].

### 4.3. High-Resolution Melting Analysis

A set of 7 SNPs was selected based on the study by Futema et al. to determine the patients’ PRS [17]. *APOE* SNPs rs7412 and rs429358 were determined using NGS or TaqMan probes. The SNP rs6544713 (nearest gene *ABCG8*) was substituted with SNP rs11220462 (nearest gene *ST3GAL4*) and rs1800562 (nearest gene *HFE*) because a suitable pair of primers could not be designed for high-resolution melting (HRM)-based genotyping of rs6544713. Using primer 3, primer pairs were generated to amplify 100 bp regions surrounding the targeted SNP [49,50]. In cases where an adequate primer pair could not be found, the targeted region was extended. To determine primer specificity and to ensure no other SNPs were present in the amplicon, Primer-BLAST and in silico PCR tools were used [51,52]. Selected primer pairs were then analyzed using the Oligo analyzer tool to avoid primer secondary structures, self-dimerization, or heterodimerization [53]. Delta G values > −2 kcal/mol for secondary structures and delta G values > −5 kcal/mol for primer dimers were considered acceptable. Finally, the hypothetical amplicons were checked using uMELT [54]. HRM primer sequences are represented in Table 3. Each sample underwent two reactions: the first reaction included primers for *LDLR* rs6511720, *APOB* rs1367117, and *HFE* rs1800562; while the second reaction included primers for *CELSR2* rs629310 and *ST3GAL4* rs11220462. Genotyping was performed using the SYBR select master mix (ThermoFisher, Waltham, MA, USA) on the Quantstudio 5 (ThermoFisher, USA) qPCR thermocycler. All reactions were carried out in a volume of 20 µL containing 10 µL of SYBR select master mix, 20 ng of genomic DNA primers (as specified in Table 1), and nuclease-free water added to finalize the volume to 20 µL. Both reactions were performed in duplicate. Cycling was carried out under the following conditions: initial denaturation at 95 °C for 2 min followed by 20 cycles of touchdown PCR consisting of 95 °C for 15 s, 59 °C for 15 s with a reduction of 0.5 °C per cycle, 72 °C for 30 s followed by 20 cycles of 95 °C for 15 s, 49 °C for 15 s, and 72 °C for 30 s. Cycling was immediately followed by HRM analysis at increments of 0.1 °C ranging from 70 °C to 95 °C. HRM data were analyzed using the ThermoFisher (USA) high-resolution melt analysis tool.

### 4.4. PRS Calculation

The PRS was calculated using weighted sums of the 7 genotyped SNPs based on the study by Futema et. al. [17] and are provided in Table 4 and Table 5.

### 4.5. Sanger Sequencing

To verify the results of HRM-based genotyping, 22 samples representing definitive genotypes were selected for sequencing. However, the set of primers for HRM analysis was designed to be as close to the variant of interest as possible. So, to carry out Sanger sequencing, a new set of primers (Table 6) was designed using the previously mentioned tools. Samples were amplified single-plex using Amplitaq Gold 360 master mix (Thermofisher, Waltham, MA, USA). Each reaction contained 17.5 µL of master mix, primers (10 µM each) as specified in Table 4, and 35 ng of DNA and nuclease-free water to a final volume of 35 µL. Cycling conditions were as follows: 95 °C for 10 min followed by 10 cycles of 95 °C for 30 s, 64 °C for 30 s with a decrease of 0.5 °C per cycle, 72 °C for 30 s, 25 cycles of 95 °C for 30 s, 54 °C for 30 s, 72 °C for 30 s, and a final extension step of 72 °C for 5 min. Amplicons were then sequenced at the “Technysium DNA Sequencing Service”.

### 4.6. Statistical Analysis

Frequencies are shown as percentages. The non-parametric Kruskal–Wallis test was used to evaluate quantitative clinical parameters. Fisher’s exact test and Pearson χ2 analysis were employed to assess the proportions of categorical variables. The association between PRS and myocardial infarction (MI) and between PRS and LDL-C levels was examined utilizing multivariate regression analysis. The subjects in the comparison group were categorized into two groups based on their LDL-C levels, with those exceeding 4.9 mmol/L, suggesting a potential association with FH [55,56,57]. All variables for the multivariate model were selected using backward selection, resulting in a final model that included only those with significance, *p*  <  0.5.

## 5. Conclusions

The PRS can be utilized to estimate the risk of MI and to predict greater than 4.9 mmol/L LDL-C concentrations in healthy individuals. However, further research is needed to incorporate additional known risk factors and rare, high-impact genetic variants to enhance the predictive accuracy and clinical utility of PRS in assessing CVD risk.

## Figures and Tables

**Table 1:** Basic characteristics of patient and comparison groups at inclusion in the study.

Variable	Patient Group n = 93		Comparison Group n = 61		*p*-Value Between Two Medians
	Median	Min-Max	Median	Min-Max	
Age in years	68	42–96	71	45–72	0.259
LDL-C concentration (mmol/L) at time of enrolment	3.88	2.87–6.63	5.67	3.19–9.7	<0.001
	N	%	N	%	*χ*2, *p-*value
Sex Men Women	48 45	51.6 48.4	34 27	55.7 44.3	*χ*2 *=* 0.252, *p* = 0.625
Smoking Current Past	0 55	0 59.1	3 14	4.9 23	*p* = 0.0114 ^#^
Anterior STEMI	38	40.9	-	-	
Family history of ischemic heart disease	39	41.9	0	0	-
Hypertension	48	51.6	45	75	*χ*2 *=* 8.368, *p* = 0.004
Diabetes mellitus	15	16.1	0	0	*-*
Renal insufficiency	11	11.8	-	-	-
Statins	29 *	31.2 *	3 **	4.9 **	*p* = 0.0001 ^#^

*—statins at hospitalization; **—statins at inclusion in the study (year 2006–2008) ^#^—Fisher’s exact test.

**Table 2:** SNPs and their frequencies were included in the PRS calculation.

dbSNP ID	HGVS	GnomAD Freq (%)	GnomAD EU FREQ (%)	Frequency (%) in the Patient Group (n = 93)	Frequency (%) in the Comparison Group (n = 61)
rs6511720	NM_000527.5(LDLR): c.67 + 2015G > T	11.38	11.49	16.13	36.07
rs1800562	NM_000410.4(HFE): c.845G > A (p.Cys282Tyr)	5.698	7.102	4.301	4.92
rs629301	NM_001408.3(CELSR2): c.*1635G > T	74.73	77.87	75.27	55.74
rs11220462	NM_006278.2(ST3GAL4): c.−61 + 17755G > A	12.41	13.77	43.01	26.23
rs1367117	NM_000384.3(APOB): c.293C > T	29.58	33.08	56.99	59.02
rs7412	NM_000041.2(APOE): c.526C > T	7.42	7.83	15.05	19.67
rs429358	M_000041.4(APOE): c.388T > C	14.85	15.06	22.58	16.39

**Table 3:** HRM primer sequences.

Name	Sequence	Amount Added (µM)	Reaction Mix
CELSR2_rs629301_F	TTGTACAGTTTGGTTGTTGCTG	2.5	2
CELSR2_rs629301_R	TACCACACAGAAGCGGACAG	2.5	2
APOB_rs1367117_F	CAGGGTTGAAGCCATACACC	15	1
APOB_rs1367117_R	TCTCAGGTTGAGCTGGAGGT	15	1
ST3GAL4_rs11220462_F	AGCGATGCTATCCGATGAAC	7.5	2
ST3GAL4_rs11220462_R	CAACTCCACACACCCAACAC	7.5	2
LDLR_rs6511720_F	TCACCAATCAACCTCTTCCTT	16.25	1
LDLR_rs6511720_R	GCCTTGCCTAAGACTTCCT	16.25	1
HFE_rs1800562_F	CTGGATAACCTTGGCTGTACC	3.75	1
HFE_rs1800562_R	GATCACAATGAGGGGCTGAT	3.75	1

**Table 4:** HRM-type SNPs and the weight used for calculations.

dbSNP rs ID	Nearest Gene	Reference Allele	Risk Allele	Weight for Score Calculation
rs629301	*CELSR2*	G	T	0.15
rs1367117	*APOB*	G	A	0.1
rs11220462	*ST3GAL4*	G	A	0.05
rs6511720	*LDLR*	G	G	0.18
rs1800562	*HFE*	G	G	0.057

**Table 5:** *APOE* haplotype’s weight used for calculations.

*APOE* Haplotype	Weight for Score Calculation
ε2ε2	−0.9
ε2ε3	−0.4
ε2ε4	−0.2
ε3ε3	0
ε3ε4	0.1
ε4ε4	0.2

**Table 6:** Sanger sequencing primers.

Name	Sequence
LDLR_rs6511720_seq_F	TGCCACTCAGTTTTACAAAAGAA
LDLR_rs6511720_seq_R	TGGAGGAAAACATCAGGGGT
HFE_rs1800562_seq_F	CAATGGGGATGGGACCTAC
HFE_rs1800562_seq_R	CACCCCCTAACAAAGAGCAG
CELSR2_ rs629301_seq_F	TCTCCCCTCAGCAATTCCTG
CELSR2_ rs629301_seq_R	TACCACACAGAAGCGGACAG
ST3GAL4_rs11220462_seq_F	AGCGATGCTATCCGATGAAC
ST3GAL4_rs11220462_seq_R	CAGCTTCTCTACTTCCCAGCA
APOB_rs1367117_seq_F	TGACTTACCTGGACATGGCT
APOB_rs1367117_seq_R	CCTCAATGCTCTGCTACCCT

## Data Availability

The datasets and resources generated during and/or analyzed during the current study are available from the corresponding author upon reasonable request.
